# Bioprospecting of the novel isolate *Microbacterium proteolyticum* LA2(R) from the rhizosphere of *Rauwolfia serpentina*

**DOI:** 10.1016/j.sjbs.2021.10.038

**Published:** 2021-10-22

**Authors:** Naushin Bano, Saba Siddiqui, Mohammad Amir, Qamar Zia, Saeed Banawas, Danish Iqbal, Roohi

**Affiliations:** a Department of Bioengineering, Integral University, Lucknow 226026, India; b Integral Institute of Agricultural Science and Technology, Integral University, Lucknow 226026, India; c Health and Basic Science Research Centre, Majmaah University, Al-Majmaah 11952, Saudi Arabia; d Department of Medical Laboratory Sciences, College of Applied Medical Sciences, Majmaah University, Al-Majmaah 11952, Saudi Arabia; e Department of Biomedical Sciences, Oregon State University, Corvallis, OR 97331, USA

**Keywords:** Actinobacteria, Secondary metabolites, GC–MS, *Microbacterium*, Antioxidant, Antibiofilm

## Abstract

The study aimed to assess the proficiency of secondary metabolites (SMs) synthesized by actinobacteria isolated from the rhizospheric soil of *Rauwolfia serpentina* for its antimicrobial and anti-biofilm activity. After morphological and biochemical identification of actinobacteria, primary and secondary screening was done for specific metabolite production. The secondary metabolites were then tested for their antioxidant, antibacterial, and antibiofilm potential. Out of 29 bacterial colonies isolated, only one emerged as a novel isolate, *Microbacterium* LA2(R). Partial 16S rRNA gene sequence of the isolate LA2(R) was deposited in NCBI GenBank with accession number MN560041. The highest antioxidant capacity of the methanolic extract the novel isolate was found to be 474.183 µL AAE/mL and 319.037 µL AAE/mL by DPPH assay and ABTS assay respectively; three folds higher than the control. These results were further supported by the high total phenolic (194.95 gallic acid equivalents/mL) and flavonoid contents (332.79 µL quercetin equivalents/mL) of the methanolic extract. GC–MS analysis revealed the abundance of antibacterial compounds; where, n-Hexadecanoic acid was found to be the major compound present with a peak of 14 min retention time (RT) and 95% similarity index. MIC value of the metabolite was noted to be around 132.28 ± 84.48 μg/mL. The IC_50_ value was found to be 74.37, 71.33, 66.28 and 84.48 μg/mL against *Escherichia coli*, *Staphylococcus aureus*, *Klebsiella pneumonia*, and *Salmonella abony*, respectively. Treatment with IC_50_ of the extract decreased the biofilm formation up to 70%–80% against pathogenic strains viz. *Escherichia coli*, *Staphylococcus aureus*, *Klebsiella pneumoniae* and *Salmonella abony*. These significant activities of *Microbacterium* sp. LA2(R) suggests that it could be utilized for antibiotic production for human welfare and in various important industrial applications.

## 1 Introduction

Actinobacteria, an advanced filamentous community of aerobic bacteria and in certain cases of facultative or obligatory anaerobic bacteria, ares well known for their delightful ability of antibiotics production (Amin et al., 2020). Bioprospecting of actinobacteria is an interesting and important area of research, as it is the most efficient and least explored class of secondary metabolites (SMs) producing bacteria with a wide variety of applications such as those related to antibacterial, antifungal, antioxidant, anticancer, antitumor and antibiofilm activities, among others (Hassan et al., 2019).

In the last decades, studies have shown that new actinobacteria species are increasingly difficult to isolate from conventional environments (Goodfellow and Fiedler, 2010, Genilloud, 2017). Therefore, researchers are changing their approach to unearth novel chemicals or drugs from new species living in eccentric environments (Li et al., 2021). Thus, the rhizosphere of numerous medicinal plants has turned out to be a potential source of advantageous microorganisms (Egamberdieva et al., 2017).

Although there are numerous bacteria in the soil, their biomass is modest due to their small size. Actinobacteria are ten-fold fewer in number but ten-fold greater in size, making them comparable in biomass to soil bacteria (Bhattarai et al., 2015). Actinobacteria found in the rhizosphere of medicinal plants have been useful for detecting novel bioactive compounds in several investigations (Amin et al., 2020, Law et al., 2020, Sharma and Thakur, 2020, Al-shaibani et al., 2021). Dynamic isolates of actinobacteria found in and around rhizosphere possess the capability to produce new inhibitory compounds (Damam et al., 2016). Despite many progress, finding a promising actinobacteria generating a broad spectrum bioactive compound with a capacity to fight against a plethora of pathogens, remains challenging (Yang and Song, 2018).

The study was aimed to isolate novel species of Actinobacteria from uncommon habitat viz. rhizosphere of *Rauwolfia serpentina*. Focus was placed on screening isolates for bioactive SMs with antibacterial, antibiofilm and antioxidant properties. 16S rRNA was used to determine the genetic diversity of the actinobacteria isolates (Sharma and Thakur, 2020). The SMs produced by an entirely new actinobacteria, *Microbacterium proteolyticum* LA2(R), demonstrated excellent anti-microbial activity against pathogenic bacteria and fungi. The SMs were also found to be quite effective against biofilms produced by MDR bacteria.

## 2 Material and methods

### 2.1 Sampling site and rhizosphere soil sample collection

The samples of soil needed for the isolation of actinobacteria’ s novel strains were obtained from rhizosphere of the medicinal plant *R*. *serpentina* at a depth of 20–22 cm using a sterile spatula. Soil samples were gathered from various places in the city of Lucknow, Uttar Pradesh, India (CSIR-CIMAP: 26.8200°N, 80.9691°E., NBRI: 26.8552°N, 80.9527°E., Botanical Garden: 28.5642°N, 77.3348°E). The samples were collected and stored in sterile plastic bags at 4 °C until use (Lee et al., 2014).

### 2.2 Pretreatment of soil samples and isolation of actinobacteria

All the collected samples of the rhizospheric soil were airdried and pretreated with CaCO_3_ to terminate the growth of the other undesirable microbes prior to the isolation, as described by Priyanka et al., (2019). Up to 10^–5^ dilutions of treated samples were prepared by serial dilution and 0.1 mL aliquot from each dilution was dispersed over different selective media; Actinomycetes Isolation Agar (AIA), Starch Casein Agar (SCA), Glycerol Asparagine Agar (GAA), Yeast Malt Agar (YM Agar) (ISP Medium No. 2, HiMedia) and Bennet’s agar, along with the cycloheximide (50 µg/mL) and nystatin (50 µg/mL), followed by incubation at 28 °C for seven days.

### 2.3 Morphological and biochemical identification of actinobacteria

The powdery white colonies of actinobacteria were isolated, and their morphological characteristics were analyzed upon inoculation of the bacteria on specific agar media (Salim et al., 2017). The Gram-positive isolates were further subjected to biochemical characterization (Yadav et al., 2018, Priyanka et al., 2019).

### 2.4 Preliminary antimicrobial screening of positive isolates

The primary screening of the isolates was performed by applying a single streak of the positive isolate (of actinobacteria against pathogenic and MDR microbes) at 90° on Mueller Hinton agar (Elbendary et al., 2018). The actinobacterial isolates were streaked as parallel lines for bacteria on the Mueller Hinton agar and for fungi on the Potato Dextrose Agar (PDA), and both plates were incubated for 4–6 days at 28 °C. The pathogenic bacterial strains, i.e., *Staphylococcus aureus* (ATCC-6538), *Pseudomonas aeruginosa* (NCIM-5029)*, Salmonella abony* (NCTC-6017), *Klebsiella pneumoniae* (NCIM-2957)*, Bacillus subtilis* (MTCC-441), and *Escherichia coli* (ATCC-25922), as well as the pathogenic fungi, i.e., *Aspergillus niger* (ITCC-545)*, Aspergillus flavus* (MTCC-277)*,* and *Aspergillus parasiticus* (MTCC-2796), were streaked at perpendicular to the earlier actinobacteria streak and then incubated at 30 °C. Zone of inhibition was calculated after incubation for 24 and 72 h in case of bacteria and fungi, respectively. Nine actinobacterial isolates were detected positive in the preliminary screening and were later referred to additional screening.

### 2.5 Secondary screening of isolates using well diffusion method

Primary screening selected isolates were further subjected to secondary screening using the well diffusion method (Messaoudi et al., 2015). The developed spores of the isolates were inoculated in 100 mL of Bennett’s broth and incubated at 28 °C in a rotary shaker at 180 rpm for ten days. On the 10th day, the agitated broth was centrifuged at 7500 rpm and 4 °C for 15 min, which separated the supernatant from the biomass. Subsequently, 50 µL of this supernatant was used for evaluating the antimicrobial activity against the different microbes as stated above. The isolates were screened based on the zone of inhibition against the tested microbes (Singh et al., 2016).

### 2.6 Molecular characterization using 16S rRNA amplification

The selected isolates from the secondary screening were further subjected to molecular characterization using 16S rRNA sequence amplification using the primers F243 (5′ GGATGAGCCCGCGGCCTA 3′) and 1378R (5′ CGGTGTGTACAAGGCCCGG 3′). Subsequently, a phylogenetic tree was created by applying the neighbor-joining DNA distance algorithm in the MEGA6 software.

### 2.7 Production of bioactive natural compounds

Secondary metabolite production from the actinobacteria was performed using Submerged State Fermentation (SmF) (Salim et al., 2017). In 1000 mL of the ISP-2 broth, 5 mL suspension of LA2(R) was inoculated, followed by incubation in a rotary shaker at 180 rpm for 20 days. After fermentation of the broth, centrifugation was performed at 7500 rpm and 4 °C for 15 min. The supernatant obtained was combined in a 1:1 ratio with ethyl acetate and mixed vigorously in a shaker at 200 rpm for 5 h. Using a separating funnel, the solvent phase was separated from the aqueous phase and subjected to rotary vacuum evaporation at a water bath temperature of 45 °C and 200 rpm to eliminate the solvent and obtain the extract in crude form. The obtained crude extract was evaluated and mixed with 1 mL methanol to form the stock solution and was stored for further use (Janardhan et al., 2014).

### 2.8 Identification of bioactive compounds by gas chromatography mass spectrometry (GC–MS)

Bioactive compounds produced by Actinobacteria were characterized with the help of GC–MS (Shimadzu QP-2010 Plus with Thermal Desorption System TD 20) (Nandhini et al., 2015). The compounds were distinguished using a GC–MS method in which 0.2 mL of a 1 mg/mL stock solution diluted in chloroform was injected into the column at a flow rate of 1 mL/min. The compounds were identified with the help of the list of similar compounds available in National Institute of Standards and Technology (NIST), whereby molecular weight (MW) and structure of the compounds identified by GC–MS were matched against those of substances in the database (Kumari et al., 2019).

### 2.9 Determination of the antioxidant potential of bioactive secondary metabolites

#### 2.9.1 DPPH assay

The free radical scavenging behavior of each fraction was measured using the 1,1-diphenyl-2-picrylhydrazyl (DPPH) assay following published protocol, with minor alterations (Dholakiya et al., 2017). The proportion of DPPH scavenging by the extracts was calculated using the formula:

(1) $$DPPH\;\left(\%\right)=\frac{Absorbance\;of\;control-Absorbance\;of\;sample}{Absorbance\;of\;control}\times 100$$

#### 2.9.2 ABTS assay

The ABTS [Azino-bis (3-ethylbenzothiazoline-6-sulfonic acid)] assay was used for measuring the activity of radical scavenging according to the process reported earlier with few modifications (Siddharth et al., 2020). ABTS was dissolved at a concentration of 7 mM in appropriate buffers (pH 7.4 and 9.0). The ABTS radical cation (ABTS+) was prepared by mixing ABTS stock solution with 2.45 mM potassium persulfate (internal concentration) and storing the mixture at room temperature for 12–16 h before use (Ishtikhar et al., 2015). The SMs extract, the ethyl acetate extract, and the methanolic extract at the concentrations of 734.97 µg/mL were mixed with ABTS and then incubated at 37 °C in the dark for 30 min. The absorbance was measured at 415 nm, and the percentage inhibition of ABTS + radicals was analyzed using the formula:

(2) $$ABTS\;\left(\%\right)=\frac{Absorbance\;of\;control-Absorbance\;of\;sample}{Absorbance\;of\;control}\times 100$$

#### 2.9.3 Total phenols and total flavonoids content

The total phenolic content (TPC) was measured with the Folin-Ciocalteu’s reagent method (Monowar et al., 2019), with slight modification. Gallic acid was utilized as a standard. The perceptible change in the color of the solution to dark blue suggested the existence of phenolic compounds (Mangzira Kemung et al., 2020). The total flavonoid content (TFC) was measured using the Aluminium chloride (AlCl_3_) colorimetric technique, with minor amendments (Monowar et al., 2019). Quercetin was used as the standard. The complete experiment was repeated three times to ensure accuracy, and the result were expressed as mean ± standard deviation (SD) (Panche et al., 2016).

### 2.10 Minimum inhibitory concentration (MIC) and IC_50_ value determination

The MIC values were determined using the microdilution method in 96-microwell plates with three replicates (Wypij et al., 2018, Contreras-Lynch et al., 2017). The lowest bioactive metabolite concentration that exhibited substantial antimicrobial activities in opposition to tested microbes was accepted as the MIC. The same procedure was applied for the determination of the IC_50_ values of the active actinobacterial extract of LA2(R). The IC_50_ value was defined as the smallest proportion of the methanolic extract of *Microbacterium* LA2(R) at which the growth of microorganisms was inhibited by 50% (Baker et al., 2020). The antibacterial efficacy of the methanolic extract of *Microbacterium* LA2(R) was compared to that of pure drugs by evaluating their IC_50_ values against *E. coli*, *S. aureus*, *K*. *pnuemoniae,* and *S. abony*. Methanol was used as a control and the bioactivity of extracts was reported based on the MIC values. Gentamicin was also utilized as a positive control.

### 2.11 Antibiofilm activity of the methanolic extract of *Microbacterium* LA2(R)

According to the reports of National Institute of Health and the Center for Disease Control and Prevention, approximately 65–80% of all infections occur due to biofilm-forming microorganisms (Jamal et al., 2018). LA2(R) has a unique capability to disrupt the biofilms of several pathogenic strains of bacteria. The biofilms of *E*. *coli*, *S*. *aureus*, *K*. *pneumoniae,* and *S*. *abony* were produced using the method reported earlier, with minor alterations (Mangzira Kemung et al., 2020). Biofilms viability was determined using the crystal violet colorimetric assay (Singh and Dubey, 2020). Percent attachment was calculated using the following equation:

(3) $$Attachment\;\left(\%\right)=\frac{Absorbance\;of\;sample}{Absorbance\;of\;control}\times 100$$

The equation below was used to calculate the percent inhibition of the biofilm.

(4) $$Biofilm\;percent\;inhibition=\left(Control\;{OD}_{490}-Test\;{OD}_{490}\right)\times 100$$

### 2.12 Analytical statistics

The statistical analysis was carried out with the Origin 6.0 software (US) (Alvi et al., 2017). The results are provided as the average and SD of three separate experiments. The mean differences between groups were examined using a one-way analysis of variance (ANOVA) with a post-hoc Tukey HSD test, and all tests were declared statistically significant at *p* ≤ 0.05 (Matuszewska et al., 2018, Shahinuzzaman et al., 2020).

## 3 Results

### 3.1 Pretreatment, isolation, identification of actinobacteria

**Fig. 1:**
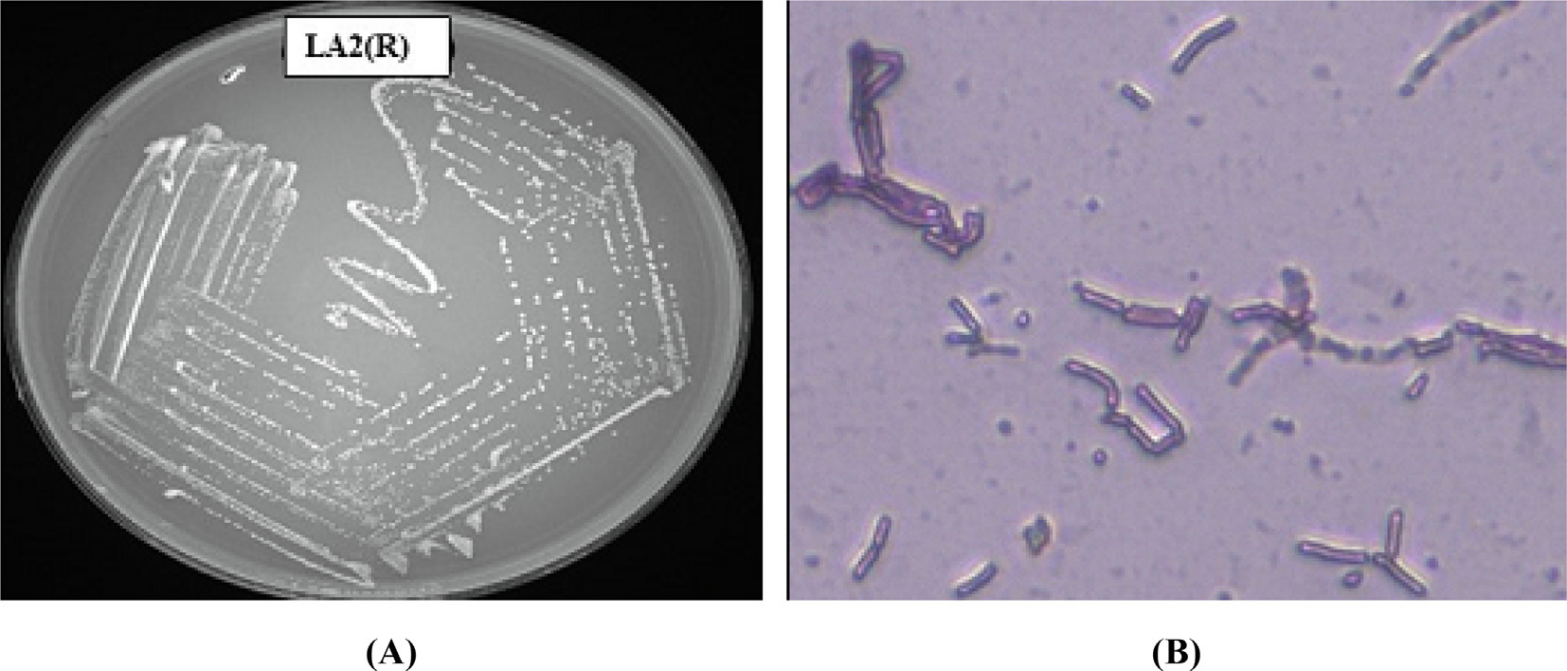
Colony morphology of the isolate LA2(R) in Actinomycetes isolation agar (left) and its microscopic view under 40× magnification (right).

Ten rhizospheric soil sample of *R*. *serpentina* were collected from different regions. The samples were air-dried, pretreated with calcium carbonate, serial diluted up to a factor of 10^–5^, and finally spread on a specific agar plate using the streak plate technique for the isolation of pure colonies. Twenty-nine pure isolates were obtained initially and were identified based on the morphological characteristics of the colonies. Out of a total of 29 isolates, only 14 tested positive for both primary and secondary antimicrobial screenings. The isolates were then subjected to further screening for biochemical characterization. The resultant morphological and biochemical characteristics of all fourteen isolates that tested positive for antimicrobial activity, are presented in Table 1 and Table 2, respectively. The color of the aerial mycelium ranged from orange to greenish to greyish-whitish chalky. Some bacterial colonies displayed greyish cream or white creamish shade. The texture of the colony varied from smooth to rough or wrinkled. Moreover, only one bacterium, LA2(O), displayed brown pigmentation. The morphological identification of the most prominent bacteria (later identified as Actinobacteria) divulged powdery white colonies, revealing spore chains and branched filaments with ariel and substrate mycelia under microscope (Fig. 1).

**Table 1:** Morphological Characteristics of the isolated actinobacterial strains.

**Isolates**	**Growth**	**Elevation**	**Surface**	**Color of aerial mycelium**	**Color of substrate mycelium**	**Pigmentation**	**Cell shape**
**LA2(R)**	Well grown	Raised	Rough	Orange	Cream	None	Log-like fragmented hyphae
**LAL**	Moderate growth	Raised	Smooth	Greyish	Faded orange	Brown	Long branching hyphae seen
**LA14N**	Well grown	Raised	Rough	Cream	Cream	None	Single spores
**LAQ**	Well grown	Raised	Rough	Cream	Cream	None	Intertwined hook- like hyphae
**LA2(O)**	Well grown	Flat	Smooth	White Creamish	Cream	Brown	Branched hyphae
**LAS**	Moderate growth	Raised	Smooth	Pale orange	Pale orangish	None	Short, fragmented rods observed
**LAW**	Well grown	Raised	Wrinkled	Greyish Cream	Cream	None	Long hyphae with spores
**LAX**	Well grown	Raised	Smooth	Mucoid greenish	Light greenish	None	Short rods
**LAK**	Moderate growth	Raised	Rough	Light yellowish red	Red	None	Spores seen
**LA3W1**	Well grown	Flat	Rough	Greyish-whitish chalky	Bright yellow	None	Grouped and single spores
**LA2A**	Well grown	Raised	Smooth	Greyish with whitish edges	White	None	Branched hyphae with spores in twos
**LA3S**	Well grown	Flat	Wrinkled	Greyish-whitish chalky	Yellow	None	Long branching hyphae
**LA 8H**	Moderate growth	Flat	Rough	White Creamish	Pale Yellow	None	Spores seen
**LA13M**	Well grown	Raised	Smooth	Greyish-whitish	Light Yellow	None	Long branching hyphae seen

**Table 2:** Biochemical characterization of the isolated actinobacterial strains.

**Strains Name**	**Indole test**	**Methyl Red**	**Voges-Proskauer**	**Citrate utilization**	**Starch hydrolysis**	**Catalase Test**	**Casein hydrolysis**	**Nitrate reduction**	**H_2_S Production**
**LA2(R)**	**+**	**+**	**+**	**−**	**+**	**+**	**−**	**+**	**+**
**LAL**	**−**	**+**	**−**	**+**	**−**	**−**	**+**	**+**	**+**
**LA14N**	**+**	**+**	**+**	**−**	**+**	**+**	**+**	**+**	**−**
**LAQ**	**+**	**−**	**+**	**+**	**−**	**+**	**+**	**+**	**+**
**LA2(O)**	**+**	**+**	**+**	**−**	**+**	**+**	**+**	**+**	**+**
**LAS**	**−**	**−**	**+**	**−**	**+**	**−**	**−**	**−**	**+**
**LAW**	**+**	**+**	**−**	**−**	**+**	**+**	**−**	**−**	**−**
**LAX**	**+**	**+**	**+**	**+**	**+**	**+**	**+**	**−**	**+**
**LAK**	**+**	**−**	**+**	**+**	**−**	**+**	**−**	**+**	**−**
**LA3W1**	**−**	**+**	**−**	**+**	**−**	**+**	**−**	**+**	**+**
**LA2A**	**+**	**−**	**+**	**−**	**+**	**−**	**+**	**+**	**+**
**LA3 S**	**+**	**−**	**−**	**−**	**+**	**+**	**−**	**−**	**−**
**LA 8H**	**−**	**+**	**−**	**+**	**−**	**+**	**+**	**+**	**+**
**LA13M**	**−**	**+**	**+**	**+**	**+**	**−**	**+**	**+**	**−**

+ Positive; **−** Negative.

### 3.2 Primary and secondary antimicrobial screening of positive isolates

**Fig. 2:**
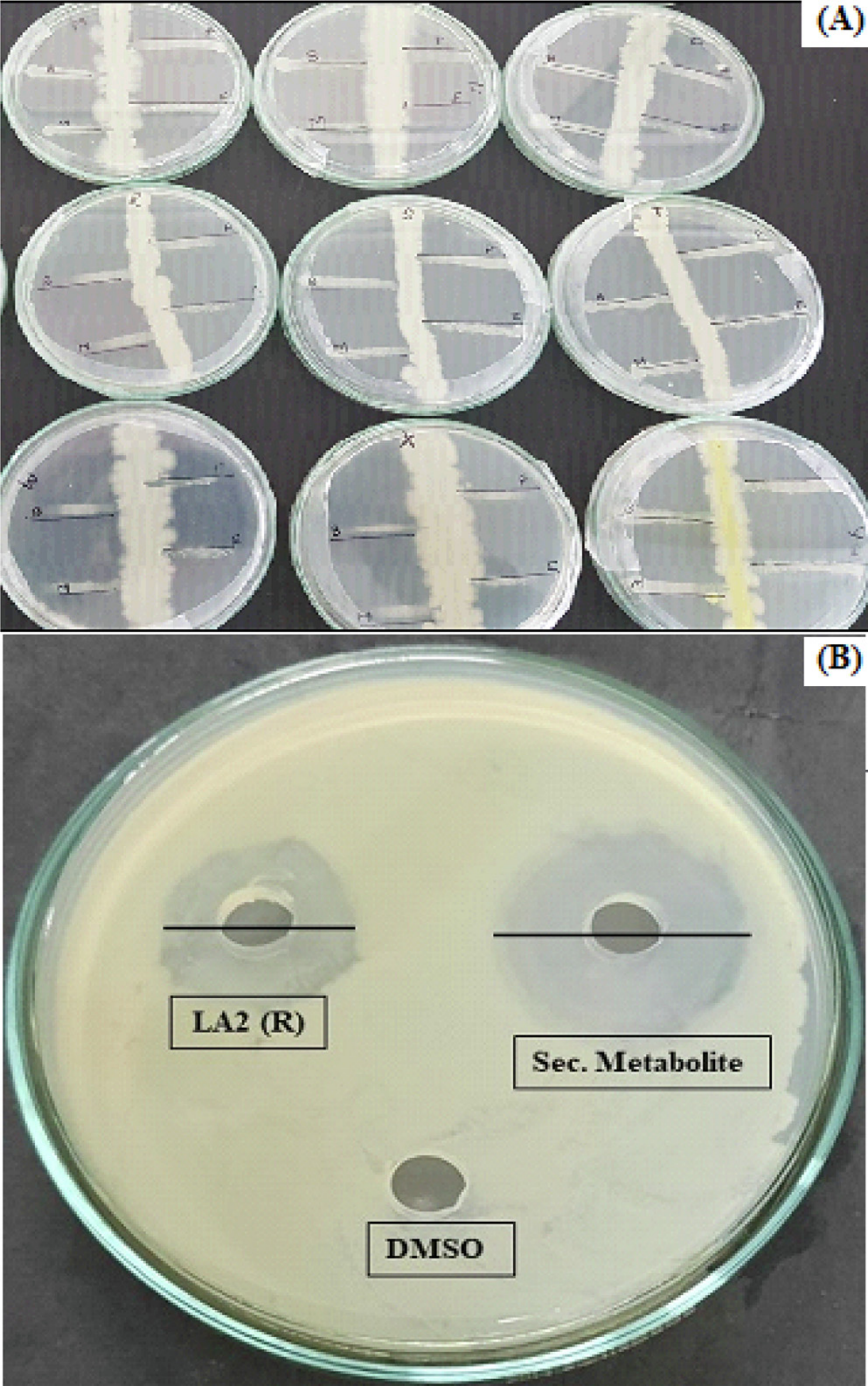
(A) Primary screening of Actinobacteria using the perpendicular streak method. (B) Secondary screening of the isolate LA2(R) using the well diffusion method.

**Fig. 3:**
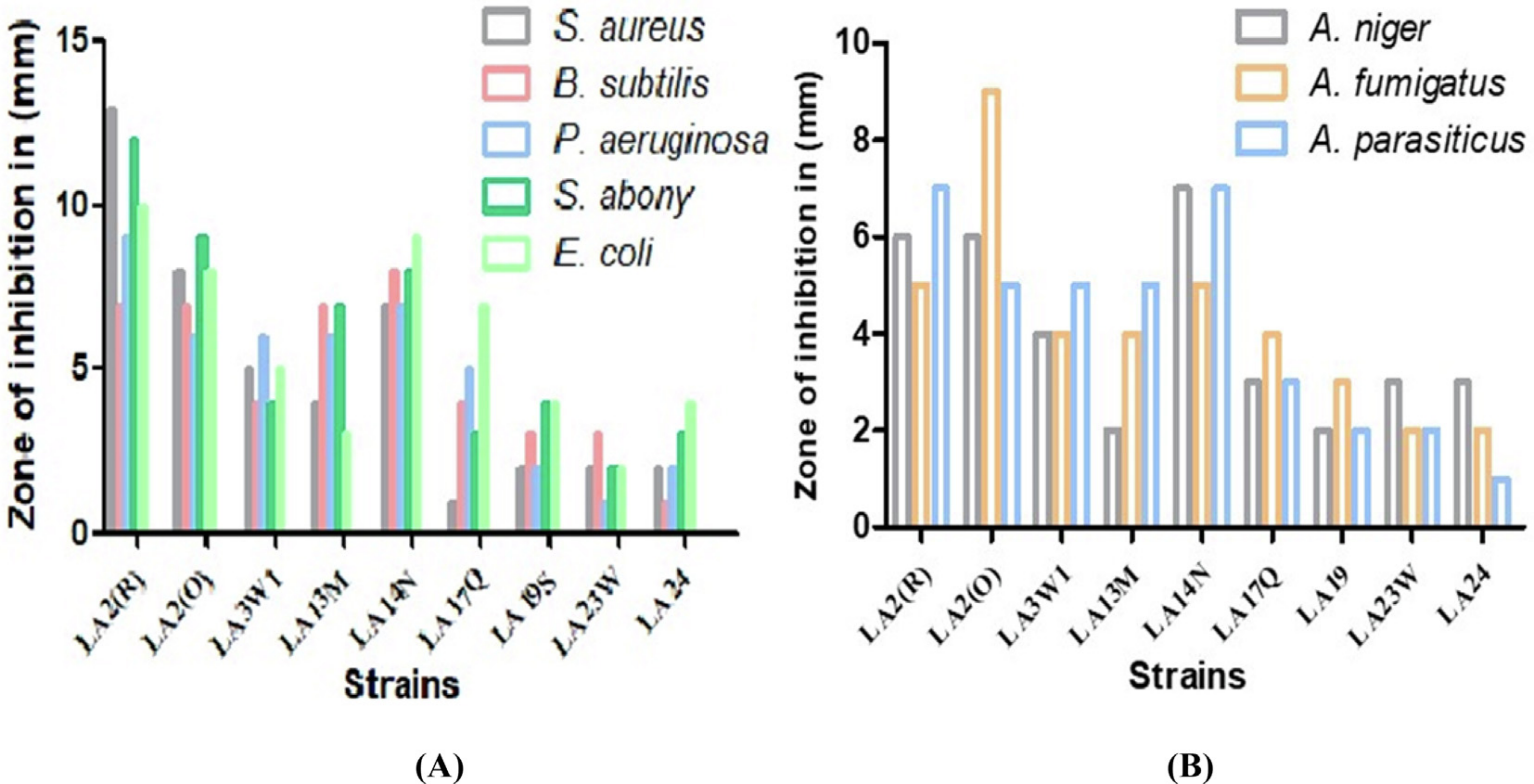
Antimicrobial screening of the positive isolates against the pathogenic (A) bacteria and (B) fungi using the well diffusion method.

Primarily, all isolates were subjected to screening for the presence of SMs by testing its antibiotic property. All 14 isolates that tested positive for antimicrobial activity were single streaked on agar for further assessment. The pathogenic bacteria i.e., *S*. *aureus*, *P*. *aeruginosa*, *K*. *pneumoniae, B*. *subtilis*, and *E*. *coli* as well as pathogenic fungi i.e., *A*. *niger, A*. *flavus* and *A*. *parasiticus* were then cross-streaked against them. The result confirmed that nine isolates exhibited significant activity against the pathogens tested (Fig. 2). Among other isolates, LA2(R) and LA2(O) demonstrated maximum antimicrobial activity. Moreover, the isolate LA2(R) showed the highest level of inhibition against *S. aureus* (Fig. 3), with an inhibition zone of ∼13 mm in diameter.

### 3.3 Molecular characterization of the isolates using 16S rRNA sequence amplification

**Fig. 4:**
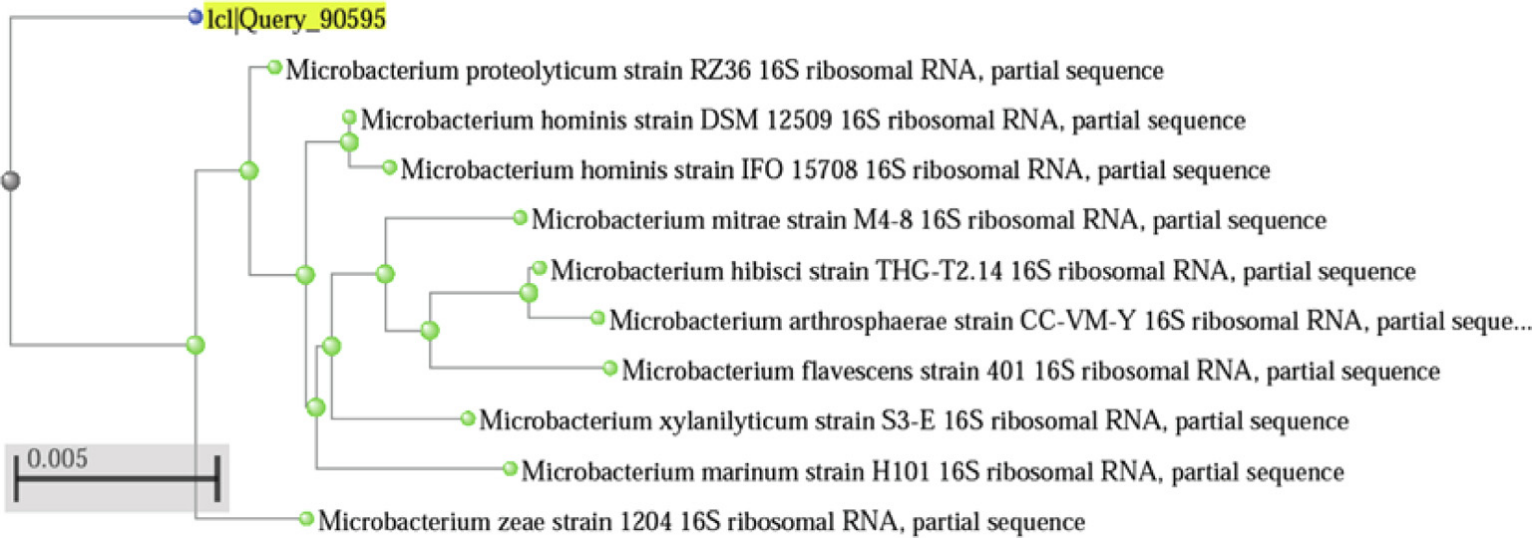
Phylogenetic analysis of isolate LA2(R) using the neighbor-joining tree method.

The identity of the selected nine isolates based on their antimicrobial activity was confirmed using 16S rRNA sequence amplification. Using the BLAST tool in the GenBank database, it has been concluded that the two isolates with highest activity belonged to the group Actinobacteria. Further, the phylogenetic analysis confirmed that one isolate belonged to the *Microbacterium* LA2(R), and other to *Streptomyces* LA2(O) genera. The *Streptomyces* species was established as *Streptomyces rochei* with 100% similarity. The *Microbacterium* species was confirmed as *Microbacterium proteolyticum* with 98.62% similarity. The partial 16S rRNA gene arrangement of isolate LA2(R) was deposited in the NCBI GenBank with accession number MN560041. Phylogenetic tree of isolate LA2(R) is depicted in Fig. 4. Since, the LA2(R) was found to be a ‘rare’ isolate, it was selected for further study.

### 3.4 Identification of bioactive compound by GC–MS

**Fig. 5:**
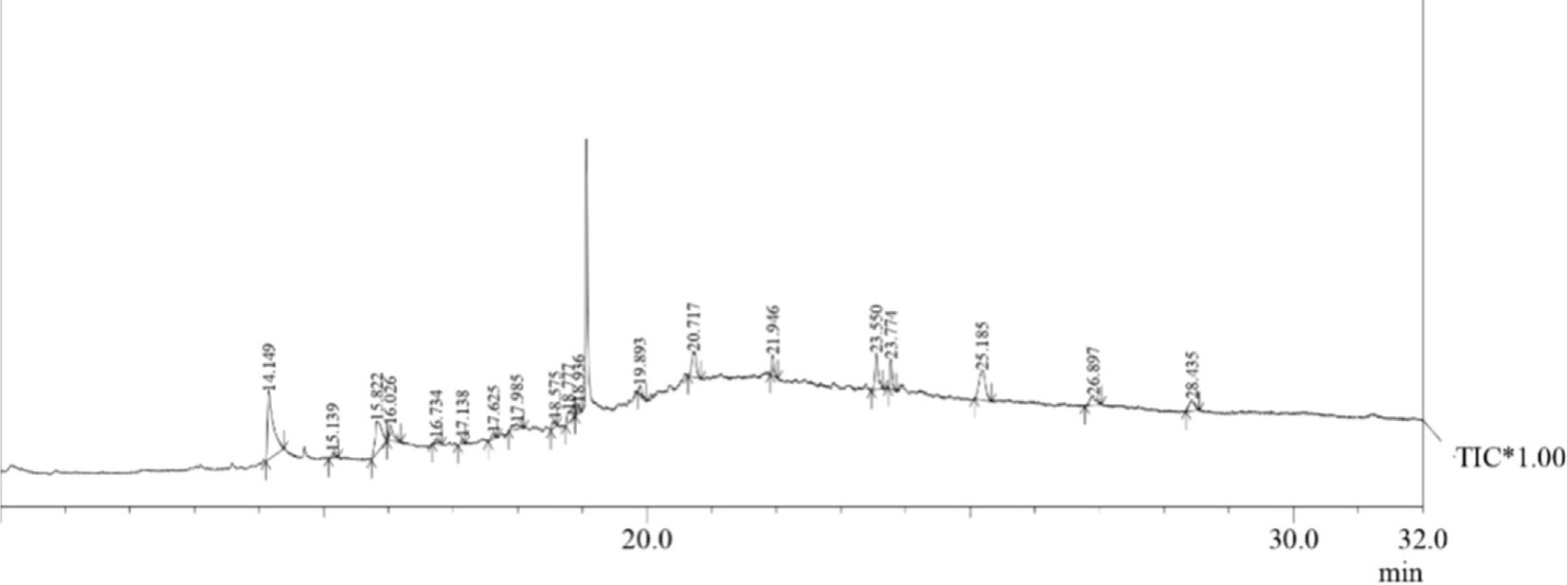
GC–MS of secondary metabolite extract prepared from *Microbacterium* sp. LA2(R).

In this study, SMs from *M*. *proteolyticum* LA2(R) were extracted using two different solvents, methanol and ethyl acetate, and were analyzed using GC–MS. Compounds present in the extract revealed the presence of four significant compounds (*cis*-Vaccenic acid, Octadecanoic acid Cholesta-3,5-diene and n- Hexadecanoic acid) based on their higher similarity index (Table 3). n-Hexadecanoic acid was the chief compound present with a peak of 14 min retention time (RT) and 95% similarity index (Fig. 5).

**Table 3:** Some prominent compounds present in Actinobacterial extract identified by GC–MS.

**Peak**	**R. Time (min)**	**Area%**	**Name**	**MW**	**Chemical formula**	**S. I.**
1	14.149	25.10	n-Hexadecanoic acid	256	C_16_ H_32_ O_2_	95
3	15.822	13.26	*cis*-Vaccenic acid	282	C_18_ H_34_ O_2_	90
4	16.026	4.36	Octadecanoic acid	284	C_18_ H_36_ O_2_	88
14	21.946	2.86	Cholesta-3,5-diene	368	C_27_ H_44_	90

### 3.5 Antioxidant potential of bioactive SMs of *Microbacterium* LA2(R)

#### 3.5.1 DPPH and ABTS radical scavenging activity

**Fig. 6:**
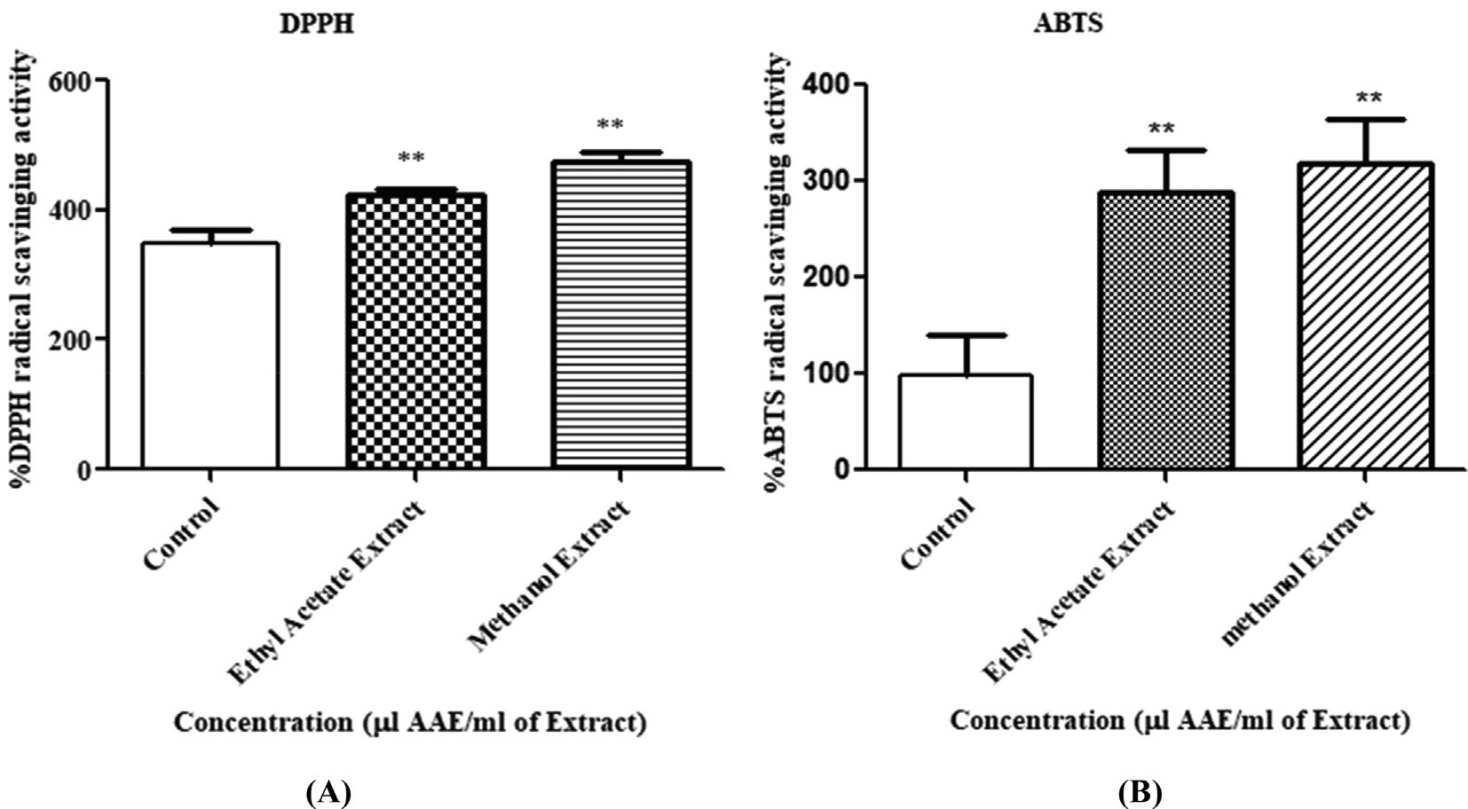
Antioxidant capacities of the pure secondary metabolite (control), ethyl acetate extract and methanol extract of Actinobacteria LA2(R). The graphs with the asterisk within the same assay exhibited no significant difference according to Tukey’s test at *p* < 0.05.

Results of the DPPH and ABTS assays revealed that the extract contained a significant amount of antioxidative compounds capable of scavenging the DPPH and ABTS radicals. Antioxidant property of the SMs extract (taken as a control), ethyl acetate extract, and methanolic extract determined in the DPPH assay were 347.493, 424.563, and 474.183 µL AAE/mL, respectively (Fig. 6A). In the ABTS assay, the values for the control (SM extract), the ethyl acetate extract, and the methanolic extract were 98.253, 287.533, and 319.037 µL AAE/mL, respectively (Fig. 6A). ANOVA revealed that the antioxidant capacities of the samples measured using the DPPH and ABTS methods displayed a substantial modification (*p* < 0.05). Tukey’s test suggested that ethyl acetate extracts of Actinobacteria differed significantly from the methanolic extracts in both the assays. The methanol extract of LA2(R) exhibited the highest antioxidant capacity than ethyl acetate extract and the pure metabolite extract.

#### 3.5.2 Total phenolic and total flavonoid content determination

**Fig. 7:**
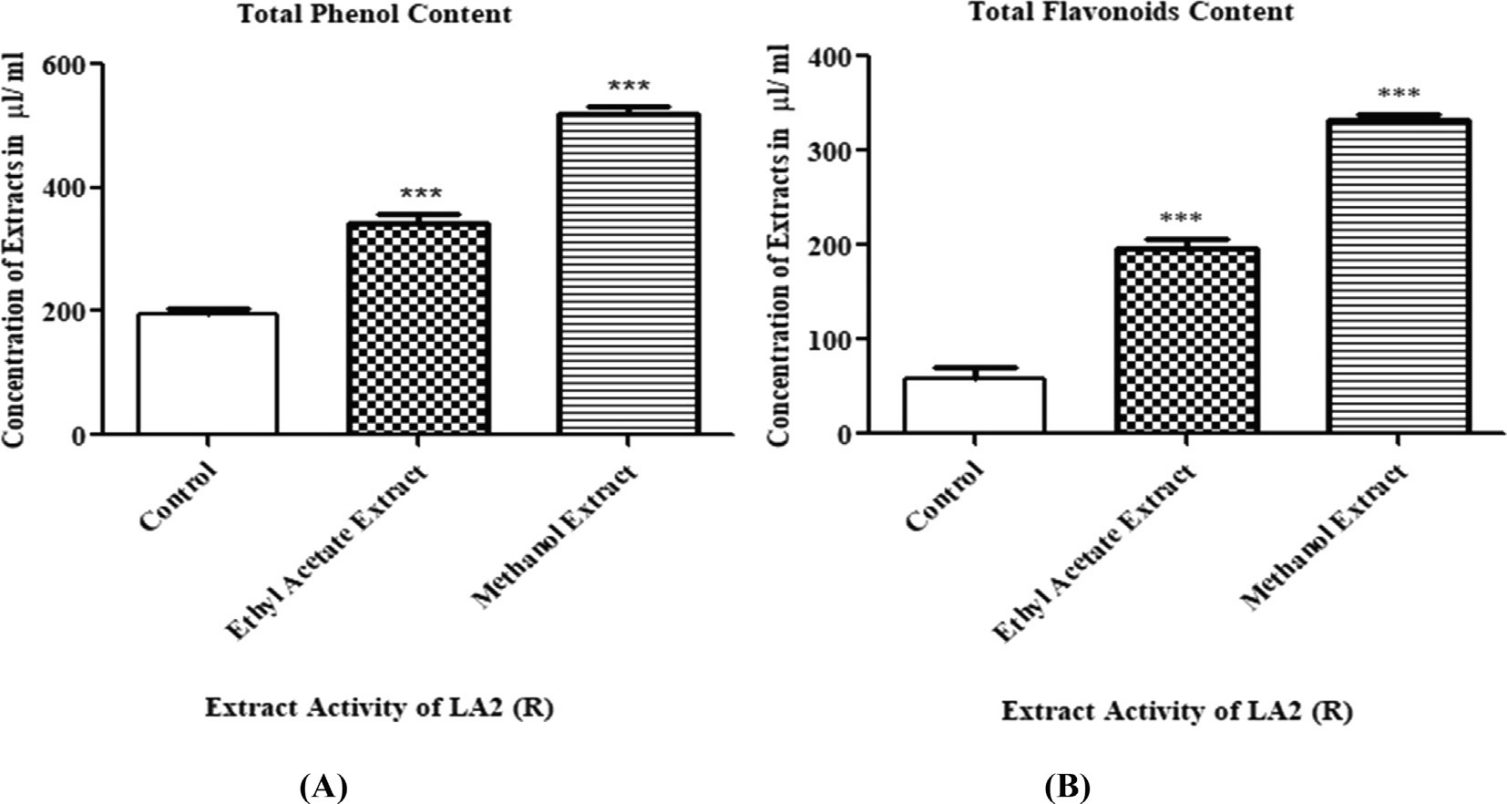
Total phenol and total flavonoid content of the pure secondary metabolite (control), the ethyl acetate extract, and methanol extract of Actinobacteria LA2(R). The graphs with the asterisk within the same assay exhibited no significant difference according to Tukey’s test at *p* < 0.05.

TPC of methanolic, ethyl acetate extracts and SM were 520.847, 342.523, and 194.95 gallic acid equivalents/mL, respectively (Fig. 7A). The TFCs in the SM extract (control), the ethyl acetate extract and the methanolic extract were 59.243 µL, 196.42 µL, and 332.79 µL quercetin equivalents/mL, respectively (Fig. 7B).

### 3.6 MIC and IC_50_ determination

**Fig. 8:**
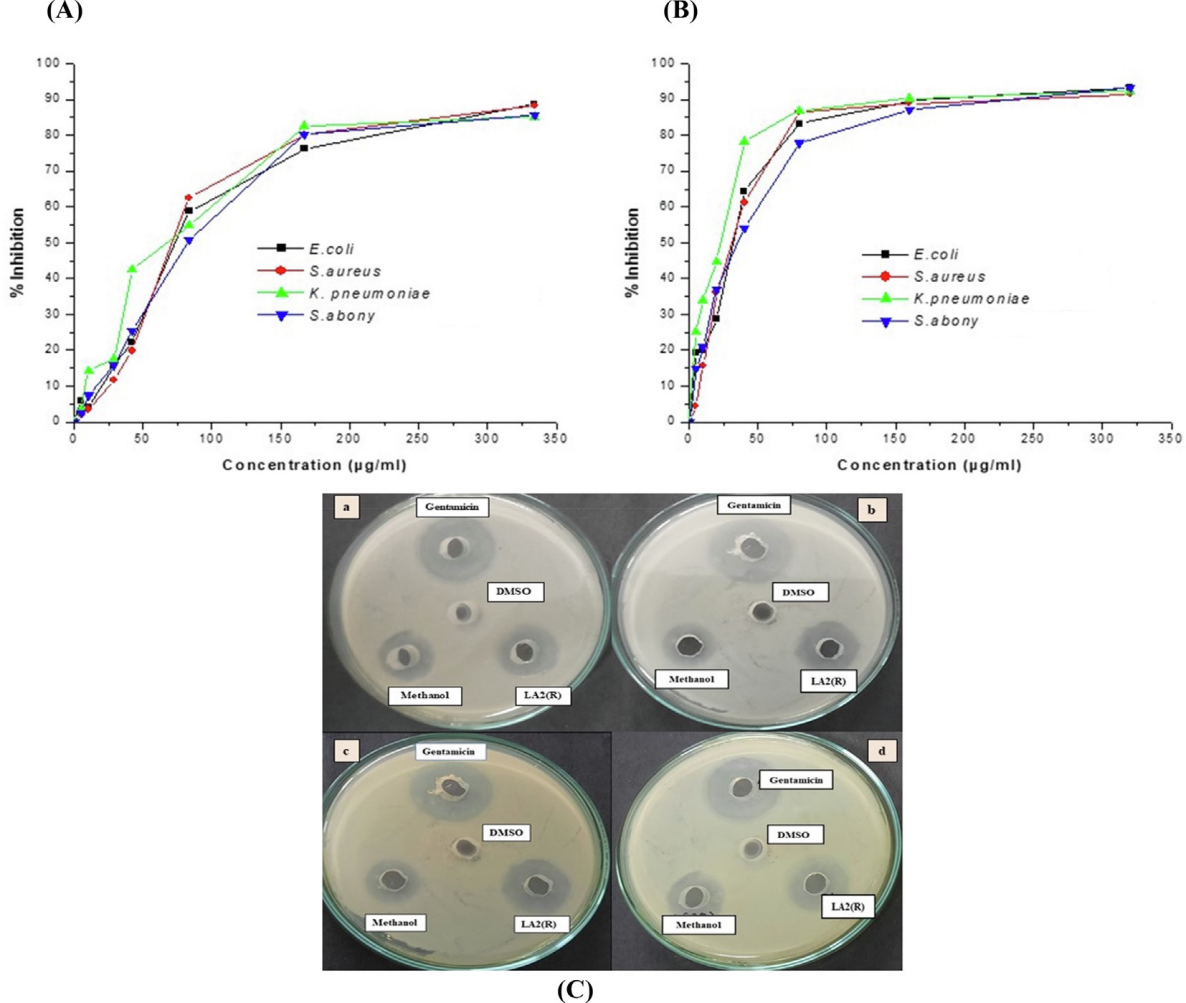
Percent inhibition of (A) methanol extract of *Microbacterium* LA2(R) and (B) Gentamicin. (C) Comparative antibacterial study of Gentamicin, DMSO, methanol as well as methanolic extract of actinobacteria against (a) *E*. *coli*, (b) *S*. *aureus*, (c) *K*. *pnuemoniae*, and (d) *S*. *abony*.

The antimicrobial activity of bioactive substances synthesized by (LA2R) isolate was established by its MIC. The inoculated plates were examined for 24 to 48 h after incubation at 37 °C (Fig. 8)*.* The MIC value of the metabolite was found to be around 132.28 ± 84.48 μg/mL. The IC_50_ value was found to be 74.37, 71.33, 66.28 and 84.48 μg/mL against *E. coli*, *S. aureus*, *K. pneumonia*, and *S. abony*, respectively. The IC_50_ value of gentamicin was found to be 31.20, 31.19, 24.11 and 35.24 μg/mL against *E. coli*, *S. aureus*, *K. pneumonia*, and *S. abony*, respectively.

### 3.7 Antibiofilm potential of the methanolic extract of LA2(R)

**Fig. 9:**
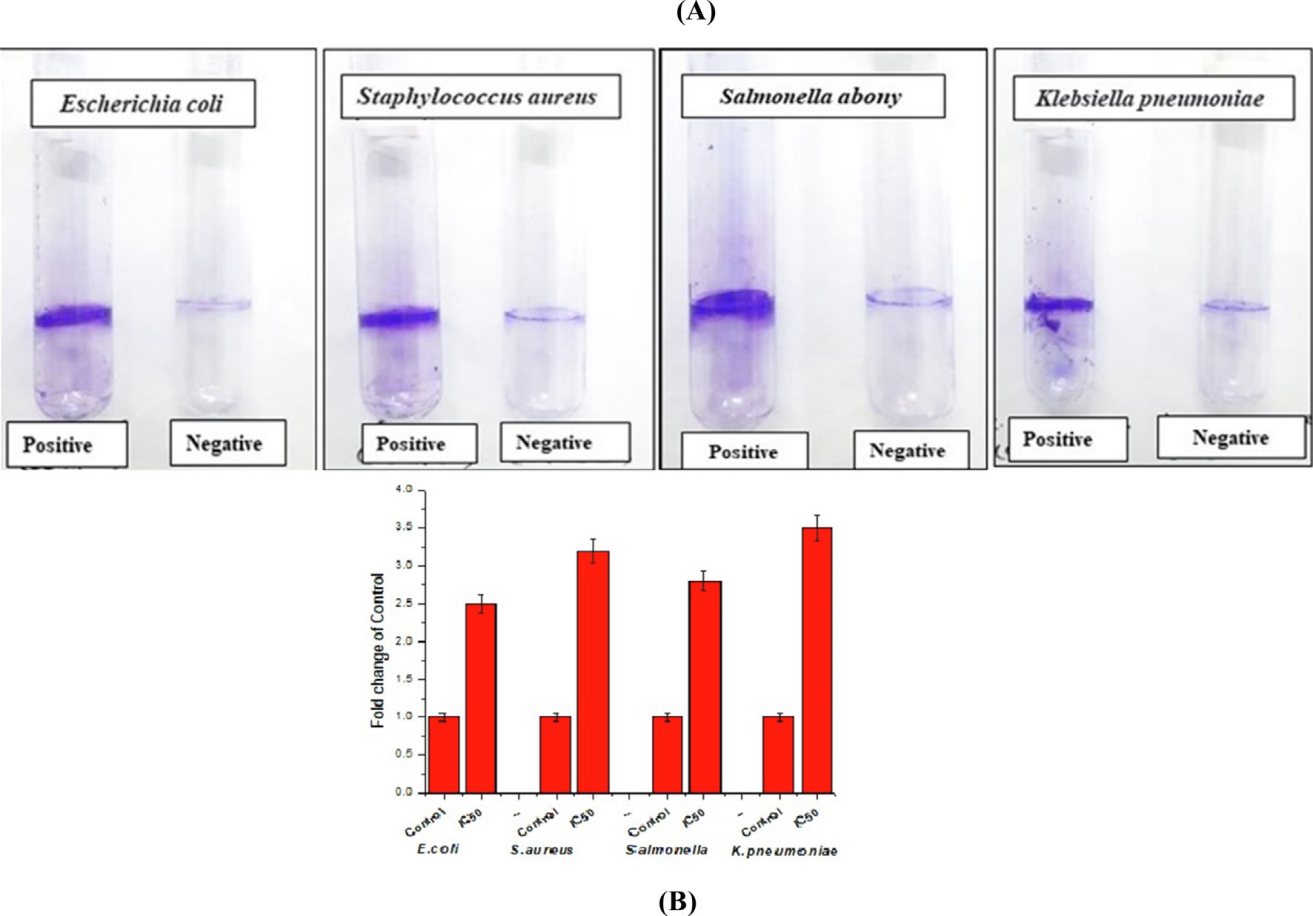
(A) Tubes depicting visible positive and negative biofilm production lining the wall against the pathogenic strains *S*. *abony*, *K*. *pnuemoniae*, *E*. *coli* and *S*. *aureus*. Test tubes labelled ‘positive’ represents a positive control. (B) The graphs representing the fold change in growth of above specified pathogenic bacteria. All experiments were conducted in triplicate, and the data were presented as mean ± SD.

The anti-biofilm potential of the methanol extract of LA2(R) was investigated using the crystal violet staining method. The test organisms were cultivated in microtiter plate with and without extracts and antibiotic. The treatment of LA2(R) extract at IC_50_ resulted in a considerable reduction (70–80%) in the biofilm formation (Fig. 9A and 9B). It was observed that methanol extract of LA2(R) served as a better antibiofilm agent than the other extract. More than threefold reduction was observed against *S*. *aureus* and *K*. *pneumoniae* (Fig. 9B).

## 4 Discussion

Primary goal of this research was to find out about the potential of the unexplored species of actinobacteria isolated from the rhizosphere of the medicinal plant, *R*. *serpentina,* with key prominence on exploiting its biosynthetic potential. Merckx et al. reported that the rhizosphere is a diverse biological environment that supports a vast spectrum of saprophytic bacteria due to the considerable intake of organic resources from plant roots and root exudates (Merckx et al., 1987, Khamna et al., 2009). It has been estimated that this narrow zone of soil can contain up to 10^11^ microbes (Berendsen et al., 2012) and harbour ∼30,000 prokaryotes (Mendes et al., 2011). Lately, rhizospheres of medicinal plants have taken center stage for investigating unconventional sources for ‘rare’ actinobacteria spp. (Golinska et al., 2015); with ‘rare’ being those species that are phylogenetically distinct from *Streptomyces* and cannot be easily isolated (Oberhofer et al., 2019). The numerous unexplored members of this group offer great prospects for new advances in the coming years.

Actinobacteria are ubiquitous Gram-positive bacteria containing high GC-content (Quintana et al., 2013). They are well documented for their incredible potential for production of natural bioactive compounds exhibiting a huge diversity of biological properties, such as antitumor, antioxidant, antibiotic and immunomodulatory activities (Barka et al., 2016, Law et al., 2020, Sharma and Thakur, 2020). Actinobacteria are mainly divided into two genera, *Streptomyces* and non-*Streptomyces* (Hui et al., 2021). Recently, the non-*Streptomyces* (including *Microbacterium* spp.), known as the ‘rare’ actinobacteria (Azman et al., 2015, Lee et al., 2014), has piqued the scientists’ interest in discovering new unprecedented bioactive compounds produced by them. Therefore, in search of a unique actinobacterial species, we collected soil samples from rhizosphere of *R*. *serpentina* from different locations at a depth of 20–22 cm. Soil samples at depth of 5–20 cm remains a fruitful source of novel actinobacteria (Jiang et al., 2016). These soil samples were utilized for isolation of rare species.

To identify actinobacteria, it is essential to develop unique prospecting technologies that are both efficient and cost-effective. For isolating ‘rare’ actinobacteria from rhizospheres, different selective methods have been exploited. Nutrient poor media with limited C- and N- supply, endorses better actinobacterial development, due to their proficient metabolic character (Oberhofer et al 2019). Initially, rhizospheric isolates were obtained by air drying the soil and pre-treating it with calcium carbonate. The spores of actinomycetes resist desiccation than most bacteria. This is advantageous as air drying activity can get rid of unwanted Gram negative bactieria, which further facilitates the isolation of actinobactierial taxa. Thus, air-dried soils can selectively isolated rare actinobacterial species (Jiang et al., 2016). Also, it has been determined that samples treated with CaCO_3_ yielded more actinobacteria (Fang et al., 2017). Further, soil samples were serially diluted up to a dilution of 10^–5^. Finally, the samples were spread on a selective media for actinomycetes and other substrates, with serial dilutions. In total, 29 probable actinobacteria isolates were retrieved from ten varied rhizospheric soil samples (data not shown). Out of 29, only 14 tested positive for secondary screening using the well diffusion method (Table 1, Table 2). These isolates were single streaked on agar and the pathogenic strains were cross-streaked against them. Only 9 isolates exhibited vital activity against the morbific microbes. The isolates were then subjected to molecular characterization to precisely identify the organisms.

Further identification and classification of isolated actinobacterial taxa as well as discrimination among other species was done using molecular approach. Thus, to develop the actinomycete phylogeny and to describe it at the species level, 16S rRNA gene-based barcoding was performed. The results of the microbial characterization revealed the presence of two actinobacteria isolates in which one novel isolate is *Microbacterium proteolyticum* LA2(R) (MN560041) and the second isolate was identified as *Streptomyces rochei* LA2(O) (Zothanpuia et al., 2015). *Microbacterium* has also been identified from the soil rhizosphere of wheat (Egamberdieva, 2012, Poomthongdee et al., 2015) as well as from rhizosphere of *Leontopodium nivale* subsp. *Alpinum* (Edelweiss), a rare alpine medicinal plant (Oberhofer et al., 2019).

Actinobacteria are efficient producers of specialized compounds with diversified structures, derived from primary-metabolites or their biosynthetic pathway intermediates (Piasecka et al., 2015). These SMs are categorized further as terpenes, steroids, flavonoids, phenolics and alkaloids (Kessler and Kalske, 2018). Furthermore, some SMs like flavonoids display robust anti-microbial action against a broad variety of pathogens (Górniak et al., 2019). In this study, extracellular SMs were extracted using ethyl acetate as well as methanol and were subjected to GC–MS analysis. All peaks in the GC–MS chromatogram matched well with the NIST library. Four significant peaks were identified based on their higher similarity index (Table 3), while several minor peaks with values below threshold were ignored. n-Hexadecanoic acid was recognized as the principal compound present in SMs with 95% similarity index (Fig. 5). N-hexadecanoic acid has also been identified in GC–MS chromatogram in several studies (Prawadika et al., 2019, Qi et al., 2019). Interestingly, GC–MS analysis of extract of a study revealed the presence of completely different components, nalidixic acid and flumequine (Das et al., 2018).

N-hexadecanoic acid (palmitic acid, PA), oleic acid, and stearic acid are typical example of fatty acids containing a carboxyl group (–COOH) and a methyl group (–CH_3_) at the two ends of an aliphatic hydrocarbon chain (Qi et al., 2019). In general, the antimicrobial properties of FA have been known for a long time (Casillas-Vargas et al., 2021). Specifically, n-Hexadecanoic acid has shown a variety of biological properties, including antioxidant, anti-inflammatory (Aparna et al., 2012), antibacterial, pesticidal, hypocholesterolemic, antipsychotic activities and acts as a hemolytic 5 alpha reductase inhibitor (Abubakar and Majinda, 2016); while *cis*-vaccenic acid, octadecanoic acid and cholesta-3,5-diene possess antibacterial and antioxidant activities (Semwal et al., 2018, Kumari et al., 2019). The major isolated metabolite, PA, is the first fatty acid generated during lipogenesis (fatty acid production). Palmitate inhibits acetyl-CoA carboxylase (ACC), inhibiting additional palmitate production (https://go.drugbank.com/drugs/DB03796). Recent studies have shown that PA can inhibit the growth of pathogens (Abdel-Naime et al., 2019, Ma et al., 2021). Thus, PA have shown better antibiotic properties that helps in human welfare (Mancini et al., 2015). Therefore, we assessed the antioxidant property and antibiotic potential of LA2(R) isolate *in vitro*.

Antioxidants forage free radicals, break radical chain reactions and prevent oxidative damage. Actinobacteria are well known for their antioxidant abilities (Janardhan et al., 2014). Lately, Larasati et al. observed the antioxidant property of actinobacteria in mangosteen peel (Larasati et al., 2020). In our case, the methanolic extract of LA2(R) demonstrated potent ability to reduce the DPPH and ABTS radicals, implying that the extract possessed noteworthy antioxidant property (Fig. 6). The methanol extract also showed good amount of TPC and TFCs as compared to ethyl acetate extract (Fig. 7). Our result is in strong agreement with a previous report that demonstrated a linear correlation between TPC and the reducing antioxidant capacity of extracts (Naz et al., 2020). The methanol extract of LA2(R) exhibited the highest antioxidant capacity compared to its ethyl acetate counterpart. Differences in antioxidant activities could be due to the use of different solvents resulting in extraction of slightly different antioxidant components in each extract.

Next, we tested methanolic extract of LA2(R) for its anti-pathogenic potential. The cross-streaking antimicrobial screenings confirmed that nine out of fourteen isolates exhibited a broad spectrum antimicrobial activity against both pathogenic bacteria as well as fungi (Fig. 2). Moreover, *Microbacterium* LA2(R) and *S*. *rochei* LA2(O) demonstrated better antibiotic activity than other isolates. *Streptomyces* sp. has long been acknowledged for its antimicrobial action as most of the known antibiotics originate from this class of species. However, in our case, the *Microbacterium* sp. demonstrated far greater activity than *Streptomyces*. The LA2(R) isolate exhibited highest action against the test organisms, displaying IC_50_ ranging from 66 to 84 µg/mL. In literature, 20 isolates of actinomycetes examined by Rahman et al., (2011) exhibited antimicrobial activity, while Elbendary et al., (2018) recorded 12 isolates that showed activity against the test bacteria.

Biofilm-associated infections are hard to treat because the immune system is unable to break through the biofilms, therefore, incapable of eliminating the bacteria. Moreover, greater antibiotic resistance behavior of the pathogen makes it challenging to develop efficient anti-biofilm remedies (Lee et al., 2014, Xie et al., 2019). Actinobacteria are amongst the potential sources that could be utilized for the development of anti-biofilm drugs. Studies have demonstrated that most of the actinobacterial species exhibit anti-biofilm activity (Azman et al., 2019). Our findings demonstrated that the metabolites of *Microbacterium* sp. LA2(R) exhibit potent anti-biofilm activity that could assist in combating the biofilm resistance. The methanolic extract of LA2(R) exhibited a significant decrease of 70–80% in the biofilm formation. Highest activity was shown against *K*. *pnuemoniae*, followed by *S*. *aureus* and *Salmonella*. Lowest fold change in biofilm formation was recorded for *E*. *coli*. This is plausible as some MDR strains are very difficult to treat due to the presence of antibiotic resistance genes. It is a well-known fact that Enterobacteria produce extended-spectrum beta-lactamases (ESBL) and other enzymes that resists most antibiotics (Teklu et al., 2019).

Overall, the present study offers the introductory data on toxicity profile and the potential bioactive compounds of a extract derived from *Microbacterium* LA2(R) that could be beneficial for future studies to assist in progress of innovative, therapeutic management of biofilm associated infections. Further investigation is warranted to determine the mechanisms underlying the antimicrobial and antibiofilm activities of the secondary metabolites of actinobacteria.

## Declaration of Competing Interest

The authors declare that they have no known competing financial interests or personal relationships that could have appeared to influence the work reported in this paper.
